# Parallel evolution of gravity sensing

**DOI:** 10.3389/fcell.2024.1346032

**Published:** 2024-03-07

**Authors:** Daria Y. Romanova, Leonid L. Moroz

**Affiliations:** ^1^ Institute of Higher Nervous Activity and Neurophysiology of RAS, Moscow, Russia; ^2^ Departments of Neuroscience and McKnight Brain Institute, University of Florida, Gainesville, FL, United States; ^3^ Whitney Laboratory for Marine Bioscience, University of Florida, St. Augustine, FL, United States

**Keywords:** gravity, exoskeleton, homology, ctenophora, placozoa, cnidaria, fungi, protists

## Abstract

Omnipresent gravity affects all living organisms; it was a vital factor in the past and the current bottleneck for future space exploration. However, little is known about the evolution of gravity sensing and the comparative biology of gravity reception. Here, by tracing the parallel evolution of gravity sensing, we encounter situations when assemblies of homologous modules result in the emergence of non-homologous structures with similar systemic properties. This is a perfect example to study homoplasy at all levels of biological organization. Apart from numerous practical implementations for bioengineering and astrobiology, the diversity of gravity signaling presents unique reference paradigms to understand hierarchical homology transitions to the convergent evolution of integrative systems. Second, by comparing gravisensory systems in major superclades of basal metazoans (ctenophores, sponges, placozoans, cnidarians, and bilaterians), we illuminate parallel evolution and alternative solutions implemented by basal metazoans toward spatial orientation, focusing on gravitational sensitivity and locomotory integrative systems.

## Introduction

Omnipresent gravity affects all living organisms; it was a vital factor in the past and the current bottleneck for future space exploration. It is anticipated that all organisms can sense the direction of the gravitational field. However, smaller bacteria-sized microorganisms (<0.5–10 µm) are subject to Brownian motion (Li et al., 2008); that is, they operate under distinct microenvironment and viscosity constraints with a lack of detectable gravity sensation. In contrast, many unicellular protists (>50 µm) and multicellular organisms have developed remarkable examples of graviperception. Gravity sensitivity often co-evolves with photoreceptors as one integrative system. In many planktonic and benthic organisms, larval stages, and land organisms, positive phototropism and negative gravitropism mutually influence each other, where photoreceptors and gravity sensors are united in decision-making during behavioral choices. Quantitatively, Hock and Hader stated: “light transduction appears to possess a logarithmic transducer, whereas gravi-transduction uses a linear one” (Hock and Häder, 2006).

Apart from numerous practical implementations for bioengineering and astrobiology, the diversity of these gravity signaling systems presents unique reference paradigms to understand transitions of hierarchical homology (Minelli and Fusco, 2013) to examples of convergent evolution within integrative systems. By tracing the parallel evolution of gravity sensing, we encounter situations when assemblies of homologous modules result in the emergence of non-homologous structures with similar systemic properties. This situation presents a perfect opportunity to study complex homoplasy at all levels of biological organization.

### Gravity sensors in unicellular eukaryotes: convergence in the assembly of eukaryotic organelles

The unicellular organisms meet ultimate physical constraints in their innovations of gravity sensing within micron-scale limitations at a single-cell level. Fundamental physical and chemical forces must be ‘addressed’ by a living system to develop gravity sensors. Mother Nature provided two principal solutions, and all of them exist in protists (Häder et al., 2006; Hemmersbach and Braun, 2006; Häder et al., 2017): (i) a heavy-weight statolith with mechanoreceptive sensor(s) or (ii) a specialized organelle/intracellular structure or a whole cell cytoplasmic content that must be more massive than the surrounding microenvironment. Both ‘designs’ provide contact and pressure on other intracellular structures, including the endoplasmic reticulum and cytoskeleton to subsequently mediate gravity signaling.

Due to small sizes, most protists do not use statoliths in their gravity orientations (Hemmersbach and Braun, 2006). The popular model organisms are the flagellate *Euglena* (Häder and Hemmersbach, 2022), the green alga *Chlamydomonas* (Kam et al., 1999; Sineshchekov et al., 2000; Yoshimura et al., 2003; Roberts, 2006), and the ciliate *Paramecium*. As summarized below, a whole cell and/or its 3D geometry might function as a sensor.

*Euglena gracilis* (∼35–60 μm) lives in freshwater habitats and ‘young’ show positive gravitaxis, but older cells show negative gravitaxis (Häder et al., 2017). Here, a whole cell content is denser than the surrounding water and acts as the statolith, providing pressure on mechanosensitive ion channels with Ca/calmodulin/cAMP signaling associated with one swimming flagellum. Häder and others determined that in *Euglena,* gravity sensors operate at “the physical limit” [0.57-1.13 pN; see (Richter et al., 2001; Häder et al., 2017)]. Interestingly, cAMP is also involved in the photoreception of *Euglena* (Iseki et al., 2002) and complex behaviors (Muku et al., 2023).

*Chlamydomonas* is a tiny alga (∼8 μm) with negative geotaxis assigned to shape-driven orientation (Roberts, 2006); the asymmetrical morphology of the cell functions as statolith-like sensor (Häder et al., 2006). Mutants of mechanosensitive-like channels eliminated gravity orientation in *Chlamydomonas* (Yoshimura et al., 2003).

*Paramecium* (∼330 μm in length) is significantly larger than *Euglena*, and its entire body also acts as a functional statolith without specialized organelle, affecting mechanosensitive and voltage-gated calcium and potassium channels in the outer membrane (Hemmersbach and Braun, 2006). Comparable observations were confirmed using another ciliate, *Stylonychia mytilus*, with an electrophysiological detection of a gravisensory potential (Krause et al., 2010).

Morphologically and physiologically functional statoliths were discovered in representatives of the genus *Loxodes* (karyorelictid ciliates), which can be up to 1 mm in size (Hines et al., 2016). The statocyst-like organelle is the Müller vesicle (∼7 μm), unique to the family Loxodidae’s ciliates. In the cavity of the vesicle, the statolith is located at the stalk with microtubules (Fenchel and Finlay, 1986), forming and acting as a true gravisensor (Fenchel and Finlay, 1986; Hemmersbach-Krause and Briegleb, 1992; Neugebauer and Machemer, 1997; Hemmersbach et al., 1998). There are 3–60 Müller organelles per cell in different *Loxodes* species (Hines et al., 2016). In *Loxodes,* the statolith is formed from BaSO_4_, whereas in a related genus *Remanella*, it consists of SrSO_4_ (Fenchel and Finlay, 1986), suggesting parallel evolution. This intracellular statocyst allows *Loxodes* to sense gravity independently from the microenvironment density but depending on oxygen gradients (Häder et al., 2017). Interestingly, gravitaxis has also been demonstrated in one of the largest ciliates known, *Bursaria truncatella* (Krause and Bräucker, 2009). *Bursaria* does not possess an analog of the Müller vesicle, and molecular mechanisms of the gravikinesis in this species are elusive.

**FIGURE 1:**
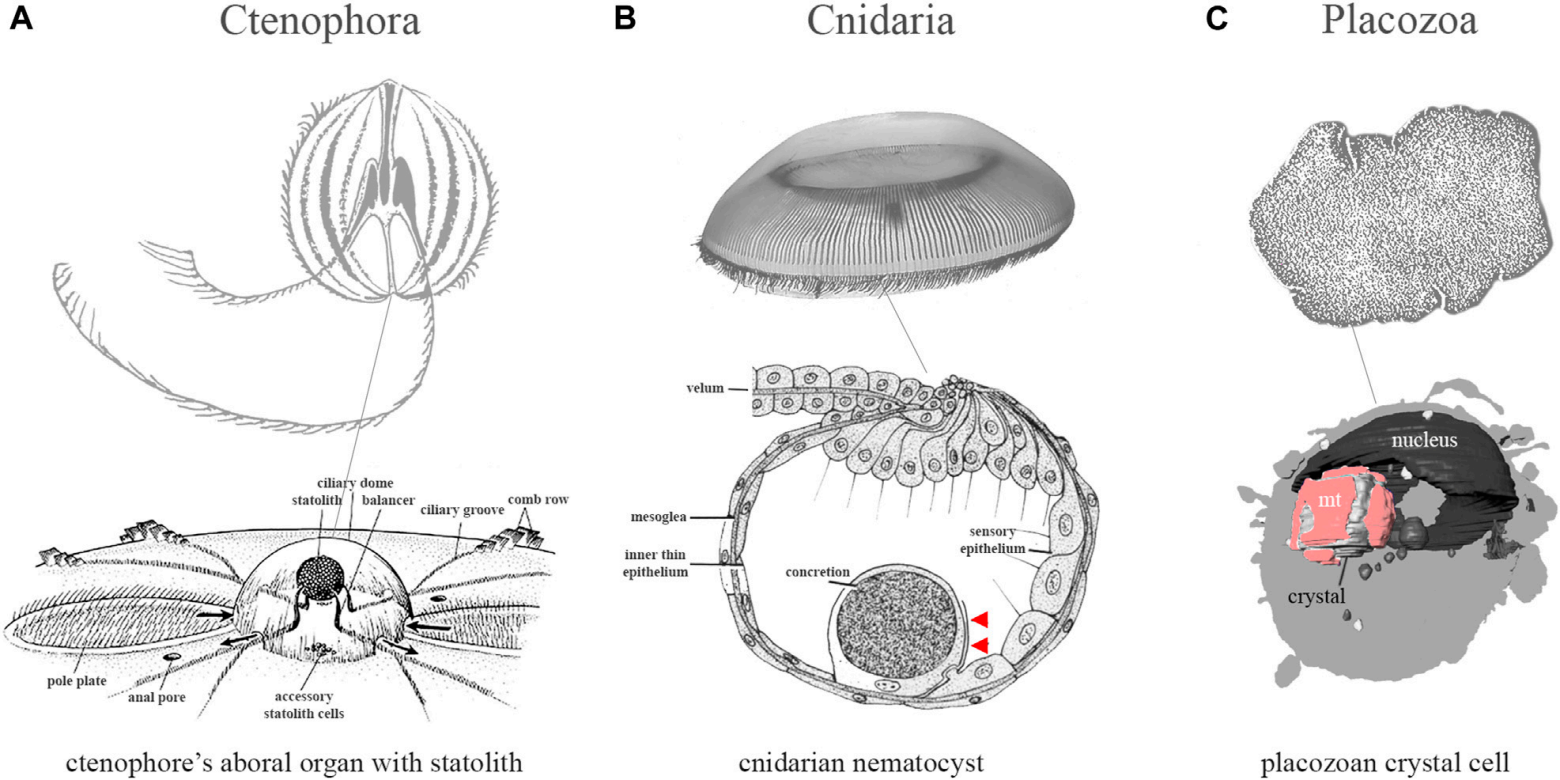
Convergent evolution of statocyst architectures in basal metazoans: Cnidaria, Ctenophora, and Placozoa. **(A)** The ctenophore Pleurobrachia (the image of the aboral organ is modified from Hyman, 1940); **(B)** The hydromedusae Aequorea (modified from Singla, 1975); **(C)** The placozoan, Tricholax adhaerens (modified from Mayorova et al., 2018).

The Müller vesicle is similar to animal statocysts, presenting an astonishing case of convergent evolution between intracellular organelle and multicellular organs. One exception in the animal kingdom is the single-cell statocyst in *Trichoplax* (Mayorova et al., 2018) with aragonite crystals (Figure 1C).

### Gravity sensing in macroeukaryotes: parallel evolution of integrative systems in complex multicellularity

A multicellular gravity structure with a heavy statolith in a specific cavity would be an ideal gravisensor in larger plants and fungi. However, this is not always the case; alternative solutions are common.

*Gravisensors in Slime molds and Fungi* are little explored, but the phenomenology is well documented (Block et al., 1986; Block et al., 1994b; Hock and Häder, 2006). In myxomycetes, the graviperception involves cAMP (Block et al., 1998). Here, nuclei and mitochondria might also work as functional statoliths (Block et al., 1986; Block and Briegleb, 1989; Block et al., 1994a; Hock and Häder, 2006).

For spore dispersal, graviresponses in basidiomycetes occur in mushroom fruit bodies (Hock and Häder, 2006). In reference species *Flammulina velutipes* and *Coprinus cinereus* [with the sequenced genome (Stajich et al., 2010)], the gravity-driven vertical orientation is fast. It takes 3–12 h, sometimes as an all-or-nothing signal (Hock and Häder, 2006), with cell nuclei as candidates for gravity-induced sedimentation (Hock and Häder, 2006; Hock and Häder, 2006).

Specialized gravity-sensing organelles have been identified in the filamentous fungus *Phycomyces blakesleeanus* (order Mucorales, Zygomycota). In this species, the tropic bending of giant single-celled sporangiophores (2 mm length and 100 μm diameter) is modulated by both light and gravity; the gravitropism is mediated through a combination of buoyant lipid globules and unique protein crystals (up to 5 μm statolith) that form within vacuoles and act as a functional single-cell statocyst (Schimek et al., 1999a; Schimek et al., 1999b). Nguyen et al. (2018) identified this octahedral crystal matrix protein (OCTIN). They demonstrated that OCTIN had been acquired from a Gram-negative bacterium by horizontal gene transfer (Nguyen et al., 2018). In contrast to bacteria constraints, giant fungal cells and acquired OCTIN mutations resulted in a new gravity-sensing function (Nguyen et al., 2018), catalyzing systemic innovations in Mucorales. Interestingly, the *Physarium* genome also contains other elements similar to prokaryotic signaling components (Schaap et al., 2015).

*Gravisensors in Plants.* Francis Darwin proposed the statolith theory of geotropism in plants (Darwin, 1903). This idea has been conceptually validated, where amyloplasts (starch-statolith as dense cytoplasmic bodies) in specialized cells mediate gravisensing and recruitment of auxin (a plant growth hormone) via its asymmetric relocalization to induce the growth of roots (positive gravitropism) or shoots (negative geotropism). The mechanisms of gravitropism include mechanosensitive channels, MAPK signaling pathways, and LAZY proteins, as the organelle-movement-triggered polarity (Häder et al., 2017; Vandenbrink and Kiss, 2019; Chen et al., 2023). Arguably, gravitropism and gravisensing evolved independently in different groups of plants, fungi, and animals.

### Gravity sensory organs in eumetazoans

Gravity receptors could be one of the earliest sense structures in animals (Vinnikov Ia, 1995; Beckingham et al., 2005) to control the orientation in 3D space. However, gravity sensors in ctenophores and cnidarians (Figures 1A,B) are not homologous structures (Moroz et al., 2014). Ctenophores are descendants of the earliest surviving lineages of metazoans, as confirmed by the most recent analyses of ancient syntenies (Schultz et al., 2023) with neural systems and the aboral organ (an analog of the elementary brain with six types of cilia (Jokura and Inaba, 2020)), evolved independently from the rest of Metazoa (Moroz et al., 2014; Moroz and Kohn, 2016). As pelagic predators (Tamm, 1982; Tamm, 2014a), they have complex and unique gravity sensing mediated by the elaborated statocyst (Horridge, 1971) with living cells (lithocytes) containing a nucleus and a large membrane-bound calcareous concretion (Krisch, 1973; Aronova, 1974; Tamm, 1982). Lithocytes are located in the aboral organ with presumed photoreceptors (Horridge, 1964; Aronova, 1979; Tamm, 2016). The statocyst is located on four balancers comprising 150–200 mechanoreceptive cilia. Balancers act as both mechanoreceptors and pacemakers (with independent duplications of mechanosensitive channels in ctenophores (Erives and Fritzsch, 2020)), mechanically transmitting signals to comb plates (Tamm, 1982; Tamm, 2014a) and contributing to the directionality of geotaxis and behaviors (Tamm, 2015). Lithocytes develop in the epithelial floor of the aboral organ (Tamm, 2014b). They are transported along the balancer cilia to form the statolith (Noda and Tamm, 2014). By ciliary surface motility, this cargo transport mechanism uniquely builds the ctenophore gravity sensing organ (Tamm, 2014a; 2015).

The most basal cnidarian lineage (Anthozoa) do not possess specialized gravity organs. In sea anemones, hair bundles on tentacles detect nearby vibrations produced by prey and discharge of cnidae to immobilize the prey (Campbell et al., 2018). However, gravity sensitivity likely exists in anthozoans (Peteya, 1973; Meroz et al., 2002), allowing them to establish and maintain growth and 3D orientations. We hypothesize that the formation of skeletons in anthozoans and polyps (Merks et al., 2004) might act as an integrative gravity-sensing system. This situation could be similar to sponges (Bart et al., 2019), which also lack specialized gravity structures.

As pelagic predators, various medusae classes have developed elaborate gravity-sensing systems with specialized statocysts connected to neural systems. Morphologically, statocysts in medusae have been studied over the century, starting with the pioneering work of the Herwig brothers (Herwig and Herwig, 1878), followed by comparative morphological analyses by Horridge (Herwig and Herwig, 1878) and Singla (Singla, 1983). Gravity sensing has also been developed in hydrozoan polyps (Ewer, 1946; Campbell, 1972; Hanson, 2023).

Scypho-medusae have been used in space research with results reminiscent of bone loss during long space missions (Spangenberg et al., 1994; Spangenberg et al., 1996). Long-term exposure to microgravity increased human bone resorption and decreased bone formation, reducing bone mineral density by approximately 2% after 1 month (Lang et al., 2004). Under similar microgravity conditions, experiments on *Aurelia aurita* ephyrae showed statolith loss from rhopalia in the space-maintained ephyrae compared to their controls (Spangenberg et al., 1994).

In Cnidaria, statocysts and lithocytes are highly diverse in forms and functions; they might not have the same development even within hydrozoans (Singla, 1975; 1983) and across medusoid classes (Horridge, 1969) such as Cubozoa (Piatigorsky and Kozmik, 2004; Mooney and Kingsford, 2016) and Scyphozoa (Spangenberg, 1991), suggesting parallel evolution in both gravity and more generalized environmental sensing. The application of calcein as a fluorescent marker allows visualization and staging of the statolith growth, and this methodology can be useful in determining the age of medusae and cubozoans (Becker et al., 2005).

In all studied species of Cubozoa and Scyphozoa the statoliths consist of calcium sulfate hemihydrate, a water-deficient phase (Becker et al., 2005). Considering the calcareous nature of statolith and environmental stressors on oceanic ecosystems (e.g., including acidification, temperature, and salinity), it would be reasonable to suggest that the elementary chemical homeostasis of gravity sensors (Morrissey et al., 2020) contribute to both short and long-term adaptations at cellular and system levels.

### Gravity sensory organs in aneural metazoans: placozoa and porifera

Porifera (or sponges) do not have statocysts or known gravisensors. These are sessile with passive filtration-feeding behaviors (Abercrombie, 1980; Bond and Harris, 1988; Stephens et al., 2013), although adult sponges can move at speeds of 1–4 mm per day (Ruppert et l., 2004). On the other hand, sponge larvae are pelagic with active locomotion (Woollacott, 1993; Leys and Degnan, 2001; Mariani et al., 2006) and explicit phototaxis behaviors (Xiang et al., 2023) but no statocysts or gravitaxis have been identified.

In contrast, placozoans are active benthic animals. Four investigated species of the genera *Trichoplax* (Smith et al., 2014) and *Hoilungia* (Romanova et al., 2021) possess specialized cell types–crystal cells with aragonite crystals and mitochondria in the middle of the cell (Figure 1C). The density of these crystal cells might vary. Carolyn Smith’s group (Mayorova et al., 2018) showed that animals’ distribution in vertical space correlates with the position of the aragonite crystal relative to the nucleus in the cell: apparently, the orientation of the nucleus remains the same, but the crystal moves relative to the cell center or nucleus if the animal changes its position by 90°. Smith’s group postulated that pressure exerted by the crystal on the plasma membrane activates mechanosensitive receptors in the membrane, which transmit a signal for position changes in space (Mayorova et al., 2018). The unique structure of the crystal cell type allows placozoan cells to maintain morphological simplicity while implementing complex behavioral acts (Romanova et al., 2021; Romanova et al., 2022). A putative nitric oxide synthase marker was detected in crystal cells and their satellites (Moroz et al., 2020), implying the involvement of the nitric oxide (NO) signaling system in space orientation and locomotion control.

Interestingly, sponges’ aragonite and silica exoskeletons have the same chemical composition as the aragonite crystal in placozoan crystal cells (Uriz et al., 2003; Uriz and Cebrian, 2006). We hypothesized the mineralization process and internal skeleton formation with subsequent cell and system polarization (Ereskovsky et al., 2007; Borisenko et al., 2019) could be ancient exaptations for gravity sensing. Spicules at developmental stages presumably play the role of growth guides, which could be a primary gravity-related function and then can be co-opted for protection as a secondary adaptive function. Future functional and single-cell analyses can help decipher the molecular blueprint of processes involved in forming 3D animal architecture and space orientation.

## Conclusion

Gravity receptions could be one of the earliest sense modifications in protists, macroscopic multicellular eukaryotic lineage, including animals (Vinnikov Ia, 1995; Beckingham et al., 2005). From the 3D space exploration perspective, trajectories of early animal evolution could drive early diversification and gravity-sensing adaptations in marine habitats with statocyst formation in larger organisms. Pelagic animals have mastered three-dimensional space, while benthic animals have predominantly expanded two-dimensional space. Predicting that statocysts and related structures are homologous across major phyletic lineages might be reasonable. However, with the advent of novel eukaryotic and animal phylogeny, this hypothesis of the common ancestry of gravity sensitivity is not correct, implying extensive convergent evolution.

Notably, the comparative analysis of gravity sensing in planktonic organisms can be used to understand better the dispersal and settlement mechanisms across species with biphasic life cycles, leveraging the neurobiology in changing marine ecosystems.
